# Draft Genome Sequences of *Erysipelothrix* sp. Strains Isolated from Stranded Septic Bottlenose Dolphins in Alabama, USA

**DOI:** 10.1128/mra.00273-22

**Published:** 2022-06-22

**Authors:** Kahylin Nesbitt, Jennifer Bloodgood, Megan M. Mullis, Alissa C. Deming, Kathleen Colegrove, Brandi Kiel Reese

**Affiliations:** a Biology Department, Oakwood University, Huntsville, Alabama, USA; b Dauphin Island Sea Lab, Dauphin Island, Alabama, USA; c Alabama Marine Mammal Stranding Network, Dauphin Island, Alabama, USA; d School of Marine and Environmental Sciences, University of South Alabama, Mobile, Alabama, USA; e Zoological Pathology Program, College of Veterinary Medicine, University of Illinois, Brookfield, Illinois, USA; University of Rochester School of Medicine and Dentistry

## Abstract

We report the genome sequences of two *Erysipelothrix* isolates from fatal cases of sepsis in bottlenose dolphins (Tursiops truncatus). The genomes were found to be most closely related to Erysipelothrix rhusiopathiae and Erysipelothrix piscisicarius. This information expands our knowledge of the genetic characteristics of this pathogen, which can affect free-ranging marine mammals.

## ANNOUNCEMENT

In February and March 2020, two bottlenose dolphin (Tursiops truncatus) carcasses (identified as 10DISL and 19DISL, respectively) were stranded off Orange Beach, Alabama. Necropsies were conducted by the Alabama Marine Mammal Stranding Network at Dauphin Island Sea Lab according to standard protocols, and paired tissue samples were collected for histological evaluation by the University of Illinois Zoological Pathology Program and for −80°C archiving. Findings indicated that both animals died of bacterial sepsis. Gram-stained tissue sections showed intracellular, rod-shaped, Gram-positive bacteria consistent with *Erysipelothrix* spp. *Erysipelothrix* may cause disease in multiple species, including humans. Sources of infection in dolphins may include environmental contamination of wounds and ingestion of fish harboring the bacteria in their mucus layer (1).

Samples of cerebrum of carcass 10DISL and spleen of carcass 19DISL, chosen because of intracellular bacteria identified during histopathology, were shipped to the National Veterinary Services Laboratories, where they were inoculated onto blood agar (R01202; Remel Products), chocolate agar (R01302; Remel Products), and MacConkey agar made in-house. Plates were incubated at 37°C in 5% CO_2_ for 18 to 24 h. Single colonies of different colony types were streaked on blood agar and incubated under the same conditions. The original plates were also reincubated. Identification was performed using a Bruker Biotyper on an Autoflex Speed matrix-assisted laser desorption ionization–time of flight (MALDI-TOF) mass spectrometer. DNA was extracted using the Promega Maxwell RSC whole-blood DNA kit. Whole-genome sequencing was performed according to established protocols using 2 × 250-bp paired-end chemistry and the Nextera XT library preparation kit on an Illumina MiSeq system, which generated 1.38 million reads for 10DISL and 1.36 million reads for 19DISL. Sequences were merged with FLASH v.2.2.0 (2), trimmed with Trim Galore v.0.6.5 (3), assembled with SPAdes v.3.15.3 (4), and annotated with Prokka v.1.11.1 within PATRIC v.3.6.12 comprehensive genome analysis (5–8). A phylogenetic tree was created in ezTree v.0.1 (9) using Prodigal (10). The amino acid sequences were compared to Pfam hidden Markov model profiles (11) using HMMER3 (12). Pfam profiles identified once were concatenated and aligned using MUSCLE (13). The alignment was used to construct a maximum likelihood tree (FastTree, JTT model, with 1,000 bootstraps) (14). Default parameters were used for all software. Average nucleotide identity (ANI) values were calculated using the JSpeciesWS webserver (15).

**FIG 1:**
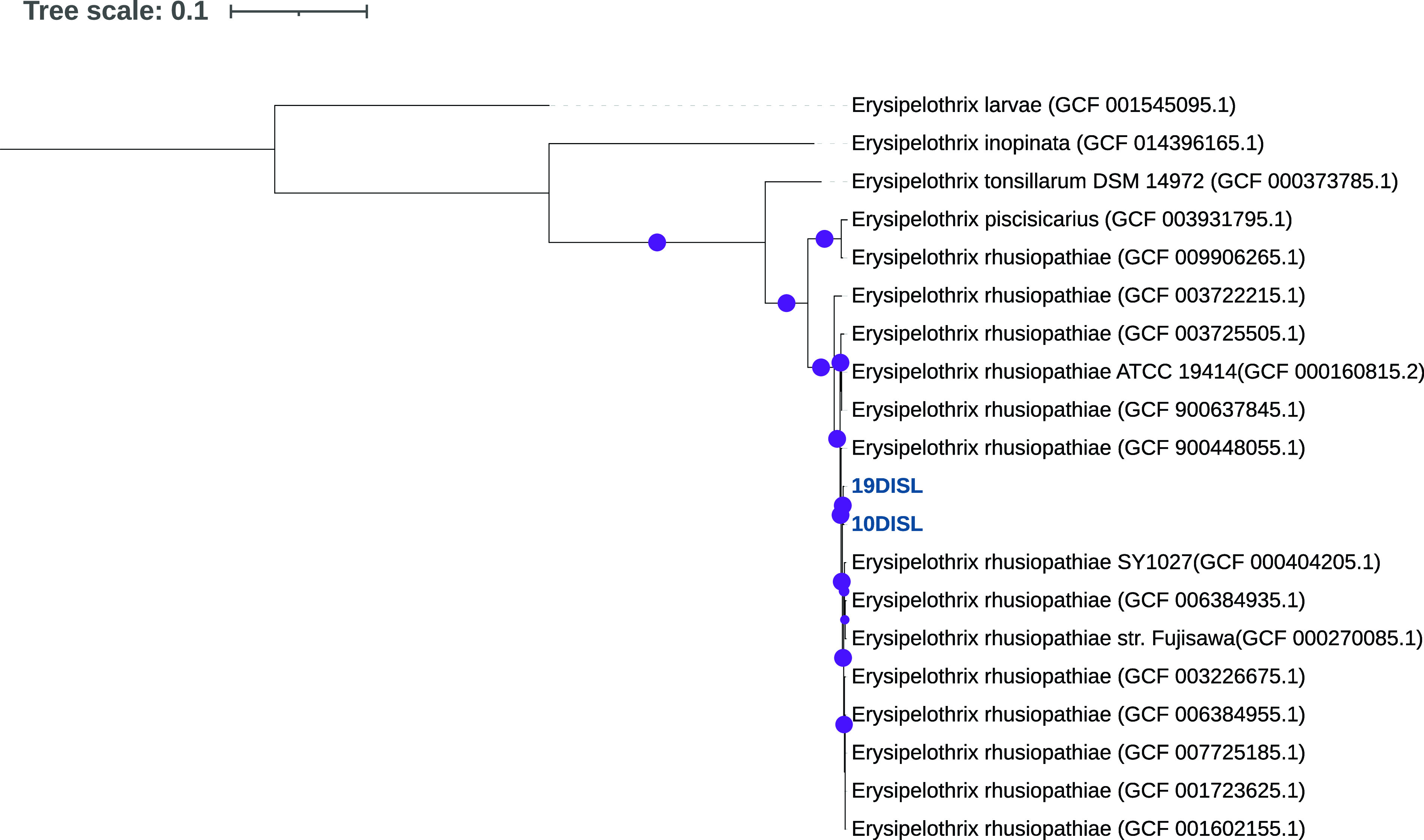
Phylogenetic tree using single-copy marker genes for whole genomes to confirm bacterial species. The purple dots represent bootstrap values greater than 70%. Numbers in parentheses are GenBank accession numbers.

Isolate 10DISL contained 96 contigs, with a G+C content of 34.46%; the *N*_50_ value was 72,029 bp, the *L*_50_ value was 7, and the genome length was 1,718,626 bp, with 1,225 protein-coding sequences, 450 hypothetical proteins, and 160× sequence coverage (Table 1). Isolate 10DISL included genes for metabolism processes (160 genes), protein processing (184 genes), energy processes (93 genes), DNA processing (66 genes), RNA processing (40 genes), stress response, defense, and virulence (53 genes), and cellular processes (51 genes). Isolate 19DISL contained 112 contigs, with a G+C content of 36.31%; the *N*_50_ value was 42,844 bp, the *L*_50_ value was 13, and the genome length was 1,807,374 bp, with 1,229 protein-coding sequences, 546 hypothetical proteins, and 146.9× sequence coverage. Isolate 19DISL included genes for metabolism processes (160 genes), protein processing (184 genes), energy processes (94 genes), DNA processing (67 genes), RNA processing (40 genes), stress response, defense, and virulence (53 genes), and cellular processes (52 genes). All annotated genes in these categories were verified as homologues using the annotation output from PATRIC, thus indicating that the gene contents across these two isolates are highly conserved. The genomes from isolates 10DISL and 19DISL were related to both Erysipelothrix rhusiopathiae and Erysipelothrix piscisicarius with 86% ANI and were related to Erysipelothrix tonsillarum with 79% ANI (Fig. 1). The isolates from this study were related to each other with 99.6% ANI, indicating that the two genomes represented a novel species.

**TABLE 1:** Assembly metrics for the two isolates

Strain identification	Isolate name	BioSample accession no.	No. of contigs	G+C content (%)	*N*_50_ (bp)	*L*_50_	Genome length (bp)	No. of protein-coding sequences	No. of hypothetical proteins	Sequence coverage (×)
*Erysipelothrix* sp.	10DISL	SAMN23594042	96	36.46	72,029	7	1,718,626	1,225	450	160
*Erysipelothrix* sp.	19DISL	SAMN23594043	112	36.31	42,844	13	1,807,374	1,229	546	146.9

### Data availability.

These whole-genome assemblies have been deposited in GenBank under the accession numbers listed in Table 1. The assembled sequences for each isolate were deposited in the Sequence Read Archive (SRA) under BioSample accession numbers SAMN23594042 (10DISL) and SAMN23594043 (19DISL).
